# Perceptions of Social Media Use to Augment Health Care Among Adolescents and Young Adults With Cystic Fibrosis: Survey Study

**DOI:** 10.2196/25014

**Published:** 2021-08-16

**Authors:** Ryan C Perkins, Rachel Gross, Kayla Regan, Lara Bishay, Gregory S Sawicki

**Affiliations:** 1 Division of Pulmonary Medicine Boston Children's Hospital Boston, MA United States; 2 Division of Pulmonary and Critical Care Medicine Brigham and Women's Hospital Boston, MA United States; 3 Division of Pulmonary and Sleep Medicine Children's Hospital of Los Angeles Los Angeles, CA United States

**Keywords:** cystic fibrosis, social media, mobile health, adherence, adolescents, young adults

## Abstract

**Background:**

For individuals with cystic fibrosis (CF), adolescence and young adulthood are times of significant vulnerability and have been associated with clinical and psychosocial challenges. Social media may offer innovative care delivery solutions to address these challenges.

**Objective:**

This study explored motivations and attitudes regarding current social media use and preferences for a social media platform in a sample of adolescents and young adults (AYA) with CF.

**Methods:**

A cross-sectional survey was administered to 50 AYA with CF followed at a large pediatric-adult CF center. The survey included questions regarding social media platform utilization, attitudes toward general and CF-specific online activities, and preferences for a CF-specific care delivery platform.

**Results:**

YouTube, Snapchat, and Instagram were the most commonly used social media platforms. AYA with CF do not report routinely using social media for health-related information acquisition, social support, or help with adherence. However, their perceptions of social media utilization and preferences for platform development suggest interest in doing so in the future.

**Conclusions:**

AYA with CF use social media and expressed interest in the development of a social media platform. Platform development will allow for gaps in health care delivery to be addressed by improving social support and adherence while augmenting current methods of health information acquisition.

## Introduction

### Background

Cystic fibrosis (CF) is the most common life-shortening genetic disease in White people in the United States [1,2]. The CF care model, which focuses on coordinated, multidisciplinary care delivery in specialized CF care centers, has contributed to improvements in clinical outcomes. CF was previously a disease with nearly uniform death in childhood but improved care has resulted in a median predicted survival age of patients of 46.2 years [1]. Current clinical guidelines for patient care recommend patients be evaluated quarterly and more frequently during the first year of life or with the illness [3-5].

Despite care delivery improvements, adolescents and young adults (AYA) with CF still face significant vulnerabilities. Studies in CF adolescents have revealed reductions in medication adherence [6], reductions in lung function despite more aggressive management and implementation of new therapies [7], increased symptom burden [8], and development or acceleration of CF-related complications [8]. Individuals with CF often face significant feelings of isolation [6,9] and are unable to congregate in typical support group structures [10] due to infection control guidelines [11]. These issues remain largely unaddressed by the current care delivery paradigm. Novel approaches to health care delivery are needed during this vulnerable time to support AYA with CF.

### Prior Work

Electronic health applications such as telehealth, mobile health (mHealth), and social media may be beneficial in addressing gaps in the current care delivery paradigm. Previously, mHealth and social media have been proposed as opportunities to engage AYA and augment care delivery [12,13]. These platforms may allow for improvements in social support, provide innovative networking opportunities for patients and their care team, and provide novel methods to improve disease self-management and adherence.

Previous mHealth studies have been carried out across diseases and were found to improve clinical outcomes, medication adherence, and self-monitoring in solid organ transplantation [14-17], essential hypertension [18,19], congestive heart failure [20], type 2 diabetes [21], and coronary artery disease [22]. However, not all have shown a clinically significant impact or improvements in adherence [23]. Within CF, a focus group study suggested implementation of a web-enabled cellphone would improve knowledge, provide social support, and result in improved adherence [24]. Another study exploring mHealth preferences noted the importance of automated assistance with disease management, improving communication with the multidisciplinary care team, and facilitating socialization with others with CF [25]. Although mHealth applications have been shown to have high acceptability [26], their long-term uptake remains problematic [13].

Social media has been used in other pulmonary diseases to understand patient experience [27], to characterize patient preferences for information acquisition [28], and to improve self-management and social support [29]. Social media has been leveraged to develop a disease-specific social network for chronic obstructive pulmonary disease to address loneliness and improve social support [30,31]. However, studies investigating social media usage in AYA with CF are lacking. Facebook has been described as a method for information dissemination by a large adult CF center in the United Kingdom [32]. Facebook, Facetime, and Instagram were described in a small qualitative study of 9 Canadian AYA with CF as a method to reduce social isolation and improve support [33]. A more recent study assessed 66 adults with CF or direct connection to CF (caregiver, significant other, immediate family member) regarding social media usage. The study found 98% of respondents used social media, with 96% of respondents using an online forum (Facebook, cysticfibrosis.com, healingwell.com). The study however had significant limitations in that it only captured adults, surveyed predominantly those without CF (39% had CF), and suffered from ascertainment bias as recruitment was performed online via email, on social media websites, and using CF dedicated message boards [34]. Larger survey studies to assess preferences for a CF-related social media platform, CF-related social media usage, perceptions regarding online CF health activities, and current social media platform usage among AYA with CF are lacking. It is crucial that patient motivations for social media usage and preferences for such platforms are explored to inform care delivery.

### Goal of This Study

The goal of this study was to characterize current social media utilization patterns and attitudes, motivations for social media utilization, and preferences for social media platform development in AYA with CF.

## Methods

### Recruitment

We performed a cross-sectional survey of AYA with CF receiving care at a large pediatric-adult CF center. Participants were eligible for enrollment if they were aged between 13-30 years and diagnosed with CF. Patients were only excluded if they had previously received a solid organ transplantation. Participants were recruited from the ambulatory CF clinic and during hospital admissions on the inpatient pulmonary service from October-December 2018. Informed consent/assent was obtained electronically, and the study was approved by the institutional review board at Boston Children’s Hospital (IRB-P0025949). The survey was administered by iPad using an online link to the survey.

### Clinical Measurements

Clinical parameters including percent predicted forced expiratory volume in 1 second (ppFEV1), hospitalizations over the last year, BMI, cystic fibrosis–related diabetes status, age, and gender were abstracted from the medical record.

### Survey Battery

The survey included 60 multiple choice questions (Multimedia Appendix 1) and explored general health/demographics (11 questions), potential components to include in a social media platform (12 questions), CF-related social media usage (11 questions), perceptions regarding online CF health activities (16 questions), and current social media platform utilization (10 questions). The topics included in the instrument were developed on the basis of previous research and scholarship surrounding social media utilization in CF [35]. The survey was piloted with 3 patients. Responses were reviewed to ensure appropriate question branching logic based on patient responses. Study data were collected and managed using Research Electronic Data Capture (REDCap) tools [36,37].

### Data Analysis

Descriptive statistics were used to analyze respondent demographics, social media utilization, attitudes/perceptions/motivations during utilization, and preferred components of a social platform. Respondent Likert scale ratings for social media platforms and attitudes during social media utilization were operationalized in the following way: “1=never” and “2=rarely” were categorized together and “3=sometimes” and “4=often” were categorized together. Additional questions for perceptions/motivations during social media utilization were operationalized as follows: “1=strongly disagree” and “2=disagree” were categorized together and “3=agree” and “4=strongly agree” were categorized together. Finally, Likert scale ratings for components of social media platforms were operationalized in the following way: “1=not at all important” and “2=somewhat unimportant” were categorized together and “3=somewhat important” and “4=very important” were categorized together. The associations of respondents’ social media utilization, attitudes/perceptions/motivations of current social media utilization, and component preferences for a social media platform with clinical parameters were assessed via chi-square test, paired *t* test, or analysis of variance, as appropriate. All analyses were conducted using STATA (version 15.1; StataCorp LLC).

## Results

During the study period, 147 patients met the eligibility requirements for inclusion in the study. Of these, 80 patients were not approached as they cancelled their appointment, were felt to be inappropriate for research by their primary pulmonologist, or finished their clinic visit before the research team could approach them. Of the 67 remaining patients, 17 declined to participate in the study. A total of 50 AYA completed the survey (Table 1).

**Table 1:** Cohort characteristics.

Characteristics of participants			Values
**Gender, n (%)**			
	Entire cohort	50 (100)	
	Males	25 (50)	
	Females	25 (50)	
Age (n=50), mean/range (SD)			19.8/13-30 (5.2)
**Racial/ethnic background,^a^ n (%)**			
	White	49 (98)	
	Latino	2 (4)	
	Black	1 (2)	
	Other	1 (2)	
**Highest education level achieved, n (%)**			
	Some high school or less	22 (44)	
	High school or General Educational Development (GED)	4 (8)	
	Some college	6 (12)	
	College/vocational degree	13 (26)	
	Professional/graduate degree	5 (10)	
**Current health status, n (%)**			
	Excellent	9 (18)	
	Very good/good	32 (64)	
	Fair/poor	9 (18)	
**Cystic fibrosis severity, n (%)**			
	Mild	24 (48)	
	Moderate	23 (46)	
	Severe	3 (6)	
Cystic fibrosis–related diabetes^b^ (n=50), n (%)			9 (18)
BMI^b^ (n=50), mean/range (SD)			21.3/15.4-35.9 (3.6)
Percent predicted forced expiratory volume in 1 second^b^ (n=50), mean/range (SD)			77.2/26-126 (23.5)
Hospitalizations^b^ (n=50), mean/range (SD)			1.4/0-8 (1.9)

^a^Respondents instructed to select all racial/ethnic backgrounds with which they identify.

^b^Abstracted from the electronic medical record.

**Figure 1:**
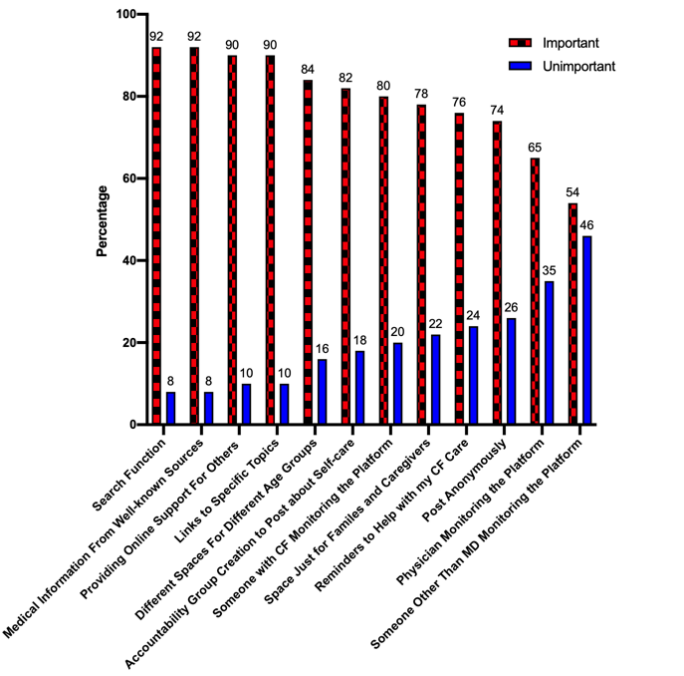
Potential components to include in a CF social media platform.

Components to include in a social platform for AYA with CF are noted in Figure 1. Respondents conveyed that a platform should include elements to help with care delivery. Overall, 44 of 49 (90%) endorsed that it was important to include a forum to provide online support for people with CF, 82% (41/50) requested accountability group creation where participants can post about self-care, and 76% (38/50) desired reminders to help with CF self-care. In addition, respondents also expressed strong support for including elements for acquisition of accurate health information. For example, 92% (45/49) wanted medical information available that came from well-known sources such as the CF Foundation and 90% (45/50) felt it was important to include links to specific CF topics.

**Figure 2:**
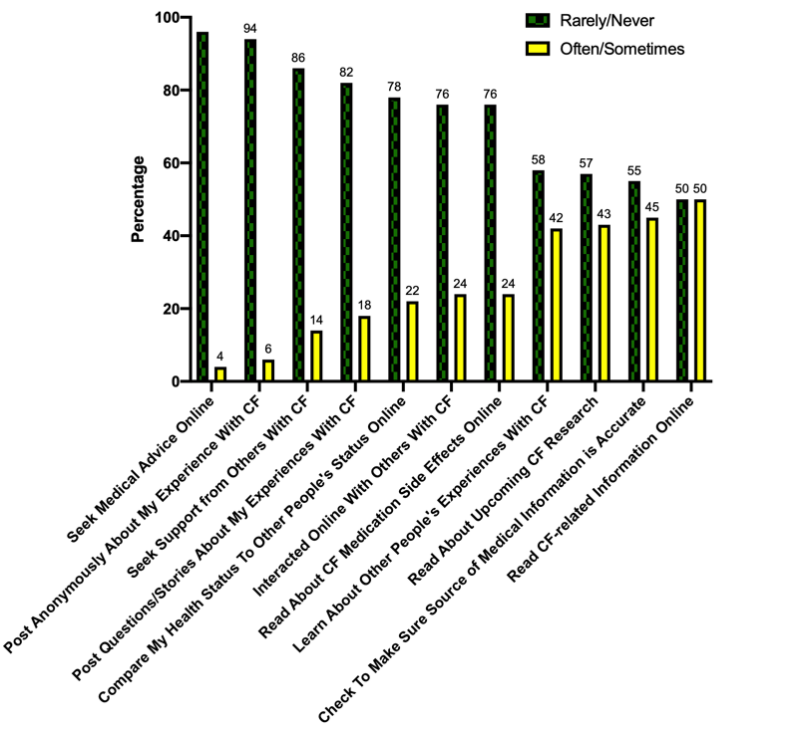
Cystic fibrosis–related social media use.

Current CF-related social media usage is shown in Figure 2. When evaluating current online health-related activities, 50% (25/50) endorsed reading about CF-related information online “sometimes” or “often.” These respondents were older (mean age of readers 22 years versus 17.6 years for nonreaders; *P*=.002) and were more likely to be female (64% female readers versus 36% male readers; *P*=.05). In addition, 42% (21/50) of respondents learned about other people’s experiences with CF online. Only 24% of respondents (12/50) endorsed interacting with other patients with CF online and even fewer (7/50, 14%) sought support from other people with CF online.

Although only a minority of respondents reported current social media use for online CF-related health activities, their perceptions regarding the potential uses for online CF-related health activities were different (Table 2). For example, only 42% (21/50) endorsed learning about other people’s experiences with CF online. However, 77% (37/48) reported they “agree” or “strongly agree” that they feel inspired by stories of other people with CF online (37/48) and 65% (32/49) noted that they wanted to motivate and inspire others with CF.

**Table 2:** Perception regarding online cystic fibrosis health activities.

Perceptions	Strongly agree/agree, n (%)	Strongly disagree/disagree, n (%)
Medical information online should come from a source like the CF Foundation (n=49)	45 (92)	4 (8)
Medical information should be from a source like a doctor (n=50)	45 (90)	5 (10)
I feel inspired by stories of other people with CF online (n=48)	37 (77)	11 (23)
It is important to me to keep my privacy online (n=50)	35 (70)	15 (30)
I want to inspire and motivate others with CF online (n=49)	32 (65)	17 (35)
Online bullying or trolling is a serious risk (n=50)	32 (64)	18 (36)
I feel motivated to perform my own self-care when I see other people with CF online (n=49)	31 (63)	18 (37)
I feel less alone when I read stories about other people’s struggles with CF (n=48)	27 (56)	21 (44)
Medical information should come from a source like pharmaceutical companies (n=50)	27 (54)	23 (46)
I feel supported by other people with CF online (n=48)	23 (48)	25 (52)
I wish I could meet other people with CF in real life after meeting them online (n=48)	22 (46)	26 (54)
I feel sad or scared when I learn about other people’s CF stories online (n=49)	13 (27)	36 (73)
I feel uncomfortable when comparing my health status to those of others with CF online (n=49)	12 (24)	37 (76)
I avoid other people with CF online (n=49)	7 (14)	42 (86)
I have met other people with CF in real life after meeting them online (n=48)	6 (13)	42 (87)
I feel less motivated to perform my own self-care when I interact with others with CF online (n=48)	2 (4)	46 (96)

Nearly two-thirds (31/49, 63%) endorsed feeling more motivated to perform their own self-care when interacting with others with CF online. In addition to the positive impact on motivation and inspiration, 56% (27/48) noted feeling less alone when they read stories about other people’s struggles with CF. Nearly half (23/48, 48%) of respondents noted feeling supported by other people with CF online. Only 14% (7/49) reported that they actively avoid other people with CF online. When considering the potential negative ramifications of online interaction, 27% (13/49) endorsed feeling sad or scared when learning about other people’s CF stories online, 24% (12/49) felt uncomfortable when comparing their health status to those of others with CF online, and 4% (2/48) felt less motivated to perform self-care when interacting with others with CF online. Regarding these perceptions, women were more likely to feel motivated to perform self-care (83% of females [20/24] versus 44% of males [11/25]; *P*=.004), feel less alone (71% of females [17/24] versus 42% of males [10/24]; *P*=.04), and endorse feeling supported by others with CF online (63% of females [15/24] versus 33% of males [8/24]; *P*=.04).

**Figure 3:**
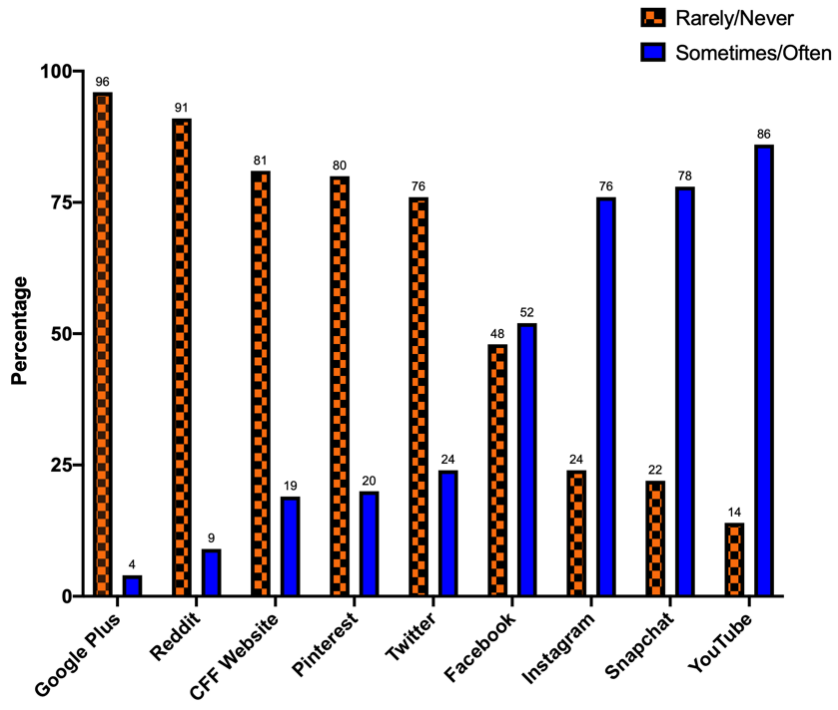
Social media platform use among adolescents and young adults with CF. CFF: Cystic Fibrosis Foundation.

Social media platform utilization among AYA with CF is shown in Figure 3. Respondents who endorsed YouTube usage were younger (mean age 19.1 years in users versus 23.8 years in nonusers; *P*=.02). In contrast, Facebook usage was higher among older respondents (mean age 21.2 years in users versus 18.1 years in nonusers; *P*=.03). Female respondents had higher usage of Instagram compared to males (92% of females [23/25] versus 60% of males [15/25]; *P*=.008).

## Discussion

### Principal Findings

This study highlights several potential avenues for CF social media platforms to improve care delivery for AYA with CF. Social support was a theme routinely identified by study participants as a potential area for exploration. Our study indicated that AYA with CF do not currently use social media for social interaction or support. This may be due to lacking a dedicated platform for interaction, not knowing others with CF, concerns about privacy of identity or medical-related information, or concerns for potential negative emotional consequences that may develop from interactions. Despite not currently using social media platforms for interaction or support, respondents generally expressed encouraging perceptions (feel more supported, feel less alone, feel inspired) regarding their attitudes after interacting with others with CF. This theme was also supported by respondents’ preferences for components in a CF social media platform. Their desire for the creation of online forums to provide support to others and the creation of accountability groups suggest participants’ perceived importance of connectedness and communication with one another. It should be noted that nearly one-fourth of respondents did experience feelings of sadness, being scared, or discomfort as a result of social interactions online. This suggests that a CF-centered platform may not be an appropriate social support structure for all, particularly those with higher risk of mental health concerns. One previous mHealth study did anticipate the need for mental health resources, medical supervision, and online moderators to provide guidance and support to address this concern [24].

There is growing evidence demonstrating the importance of social support among patients with CF and their health-related outcomes. A longitudinal survey study of 250 adults with CF explored this relationship and found fewer self-reported mental and physical health symptoms, digestive symptoms, and eating disturbances. Social support was associated with improved emotional, social, and role functioning, vitality, and body image. Those who reported more social support perceived less treatment burden and experienced better overall perceptions of their health [38]. Another cross-sectional study of 233 adults with CF highlighted the association of gender on perceived social support in adults with CF, with females perceiving greater levels of support [39]. Likewise, our study also revealed a gender disparity, with females perceiving a greater level of support, increased motivation to perform self-care, and feeling less isolated after interacting with others compared to males in our study. These studies highlight the potential role for a CF social media health platform in improving care delivery through augmenting social support and improving health outcomes.

Respondents to the survey also felt a CF social platform could be used to improve motivations for adherence. Participants generally endorsed increased motivation to perform self-care after interacting with others with CF. It is plausible that increased emotional support could lead to improved motivation among respondents and ultimately to an increase in adherence with performing self-care. Increased social interaction did not appear to have a negative reported effect on adherence. Respondents’ desires for potential components of an electronic platform (developing accountability groups, reminders to help with self-care) also suggest their interest in improving care delivery through virtual community building while also allowing for individualizing adherence assessments. The respondents’ motivations and desires are congruent with interventions proposed to improve adherence through the implementation of patient-centered treatment plans, harnessing technology and application development to increase patient motivation and virtual support, and improving the CF health service model to address individual barriers to adherence [2]. These motivations and desires suggest the possible positive implications that a CF social media health platform can have in improving care delivery through improved patient adherence.

This study indicated that AYA with CF do commonly use social media. Despite this, the majority of respondents do not currently use social media platforms for health-related information acquisition (read about upcoming research, read CF-related information online, read about medication side effects). Their perceptions about what components are important to include in a social media platform suggest their interest in health-related information acquisition online as evidenced by the perceived importance of including a search function, medical information coming from well-known sources, and links to CF-specific topics. It is unclear if participants do not currently perform these actions because they are unaware of where to acquire this information or if they do not trust social media platforms as sources of information. The latter is consistent with a cross-sectional survey of 204 AYA patients without CF in which only 25% felt social media provided useful health information [40]. The Cystic Fibrosis Foundation website provides robust information about CF, medications, and upcoming research that is evidence- and consensus-based, although our study indicates that AYA rarely use this resource.

### Limitations of Study

This study has several limitations. First, participants in our study were varied in age, with the majority of respondents under the age of 21. This age distribution may skew the cohort sample and make results less generalizable to adults with CF. However, all of the respondents would be potential users of a future social media platform. Second, though small, our study represents one of the largest samplings to date of AYA patients with CF to investigate social media. Our participants were actively recruited from a CF clinic and during inpatient admissions, which resulted in a study population more representative of the CF community at our large, urban CF center. However, these findings may not be representative of all AYA with CF.

### Future Directions

Our study suggests that although AYA with CF do not routinely use existing social media platforms for health-related behaviors, they do express interest in harnessing social media platforms for improving care delivery, accessing social support, and improving therapy adherence. The COVID-19 pandemic created psychosocial challenges for youth with chronic disease, which have implications for mental health and social supports [41]. Likewise, the pandemic also allowed for rapid-cycle digital advances in health care delivery [42] (eg, the popularization of the use of video-based applications for routine clinical communication). Many social media applications currently integrate video-based functions for users. This may allow for synchronous communication between people/care teams using videoconferencing rooms on Facebook or messaging/video calling features on WhatsApp, Facebook Messenger, or Google Duo. In addition, asynchronous communication may also have utility by posting recordings using Facebook Live or Instagram Live, or posting video stories (a feature of many social media platforms) for others as a form of encouragement or as a reminder. These features may have utility as a means for providing group-based support, facilitating accountability groups, or fostering socialization between AYA with CF and warrant further investigation. Additional considerations should be given to exploring the perceptions surrounding social media platform integration with the CF multidisciplinary team and routine clinical care, privacy concerns, and the implications of social determinants of health on social media use.

Finally, additional insight is needed to characterize baseline social isolation in this population and the desired modalities for support (individual video chats, messaging functionality, group video meetings, etc). It would be interesting to assess the use of social media and patients’ reported sense of social isolation. For example, do AYA with CF who use social media have a lower sense of social isolation compared to those who do not use social media? Are there differences based on the type of social media modality used? Does gender have an impact on the sense of social isolation? Addressing social isolation is important as a possible means to improve self-care, adherence, and health outcomes. The implications are likely relevant for other chronic illnesses.

In conclusion, our study highlights the possible utility of social media platforms as an innovative intervention for improving health care delivery, social support, and treatment adherence. Overall, gaining insight from AYA with CF during any kind of intervention development will be critical to ensure effectiveness and improving value.

## Appendix

Multimedia Appendix 1 Social media survey battery.

## Abbreviations

AYA: adolescents and young adults
CF: cystic fibrosis
mHealth: mobile health
ppFEV1: percent predicted forced expiratory volume in 1 second
