# Supplementary material for: Perceptions of Social Media Use to Augment Health Care Among Adolescents and Young Adults With Cystic Fibrosis: Survey Study
Source: JMIR Pediatr Parent. 2021 Aug 16;4(3):e25014. doi: 10.2196/25014 (PMC8406102; doi:10.2196/25014)
Supplement: Multimedia Appendix 1 [file pediatrics_v4i3e25014_app1.docx]

**Thank you for participating in this survey. This survey will provide us with some basic information about you, your Cystic Fibrosis (CF), and your opinions and patterns of using social media. *Social media* refers to any online space or website/app where the users can post comments, videos, information, or other “content” for other people to see (sites like Twitter, Facebook, Instagram, and Snapchat).**

**Basic Information**

How old are you? (drop down option from 13-30)

How do you identify your gender?

[] Male

[] Female

[] Trans*

[] Other

How would you rate your current health status?

[] Excellent

[] Very good

[] Good

[] Fair

[] Poor

How do you rate the severity of your cystic fibrosis?

[] Mild

[] Moderate

[] Severe

Please select the range of your baseline FEV1 (when you are not sick)

[] Greater than 70% predicted

[] 41-69% predicted

[] 40% predicted or less

How many times have you been hospitalized for your CF in the past 12 months?

[] I have not been hospitalized for my CF in the past 12 months

[] 1-2 times

[] more than 2 times

In the past 3 months, how often did you use the following websites?

|  | Never | Rarely | Sometimes | Often |
| --- | --- | --- | --- | --- |
| Cystic Fibrosis Foundation Website |  |  |  |  |
| Facebook |  |  |  |  |
| Google Plus |  |  |  |  |
| Instagram |  |  |  |  |
| Pinterest |  |  |  |  |
| Reddit |  |  |  |  |
| Snapchat |  |  |  |  |
| Twitter |  |  |  |  |
| Youtube |  |  |  |  |
| Other |  |  |  |  |

Please list any other social media websites/apps that you use:

**CF Related Social Media Use:** In the past 3 months, how often did you use social media in the following ways?

|  | Never | Rarely | Sometimes | Often |
| --- | --- | --- | --- | --- |
| I read CF related information online |  |  |  |  |
| I read about CF medication side effects online |  |  |  |  |
| I read about upcoming CF research online |  |  |  |  |
| I learn about other people’s experience with CF online |  |  |  |  |
| I seek support from other people with CF online |  |  |  |  |
| I post questions or stories online about my experience with CF |  |  |  |  |
| I post anonymously online about my experience with CF |  |  |  |  |
| I have interacted online with other people with CF |  |  |  |  |
| I compare my health status to the health status of others with CF online |  |  |  |  |
| I seek medical advice for my CF online |  |  |  |  |
| When I find CF related medical information online, I check the source to make sure it is accurate |  |  |  |  |

How much do you agree or disagree with the following statements?

|  | Strongly Disagree | Disagree | Agree | Strongly Agree |
| --- | --- | --- | --- | --- |
| I avoid other people with CF online |  |  |  |  |
| Medical information online should come from a source like the CF Foundation, |  |  |  |  |
| Medical information should be from a source like a doctor |  |  |  |  |
| Medical information should come from a source like the pharmaceutical companies |  |  |  |  |
| I feel *less alone* when I read stories about other people’s struggles with CF |  |  |  |  |
| I feel *supported* by other people with CF online |  |  |  |  |
| I feel *uncomfortable* when comparing my health status to those of others with CF online |  |  |  |  |
| I feel *motivated* to perform my own self-care when I see other people with CF online |  |  |  |  |
| I want to *inspire and motivate others* with CF online |  |  |  |  |
| It is important to me to keep my privacy online |  |  |  |  |
| Online bullying or trolling is a serious risk |  |  |  |  |
| I feel *less motivated* to perform my own self-care when I interact with others online with CF |  |  |  |  |
| I feel *sad or scared* when I learn about other people’s CF stories online |  |  |  |  |
| I feel *inspired* by stories of other people with CF online |  |  |  |  |
| I wish I could meet other people with CF in real life after meeting them online |  |  |  |  |
| I have met other people with CF in real life after meeting them online |  |  |  |  |

**If you could design a social media platform for teens and young adults with CF, how important are the following?**

|  | Not at all important | Somewhat unimportant | Somewhat important | Very important |
| --- | --- | --- | --- | --- |
| Being able to post anonymously |  |  |  |  |
| A search function |  |  |  |  |
| A doctor who monitors the platform |  |  |  |  |
| Medical Information from well-known sources (CF Foundation, etc.) |  |  |  |  |
| Links to specific topics (ex. Medications, insurance, travel, etc) |  |  |  |  |
| Someone other than a doctor who monitors the platform |  |  |  |  |
| At least one person with CF who monitors the platform |  |  |  |  |
| Space just for families and care givers |  |  |  |  |
| Reminders to help with my CF care |  |  |  |  |
| Different spaces for people different age groups |  |  |  |  |
| Accountability groups where you can post about your self care (ex. Remembering treatments, chest PT, etc) |  |  |  |  |
| Providing online support for people with CF |  |  |  |  |

If you have any other comments about the issues raised above, please add them here: (free text)

What is the highest grade of school of you have completed?

[] Some high school or less

[] Some college

[] High school diploma/GED

[] Vocational School

[] College Degree

[] Professional or graduate degree

Which of the following describes your current work or school status?

[] Attending school outside the home

[] Taking educational courses at home

[] Seeking work

[] Working full or part time (either outside the home or at a home)

[] Full time homemaker

[] Not attending school or working due to my health

[] Not working for other reasons

Are you Latina/Latino?

[] Yes

[] No
[] Prefer not to answer

If yes, which of the following best describes your background? Check all that apply:

[] Puerto Rican

[] Cuban

[] Mexican

[] Central or South American

[] Member of another group

Which of the following best describes your racial background? Please select all that apply:

[] White

[] Black or African American

[] Native Hawaiian or other Pacific Islander

[] Asian

[] American Indian or Alaska Native

[] Other

[] Prefer not to answer

If you chose “other” for your racial background, please specify:
